# The dynamic evolution of the characteristics of exchange rate risks in countries along “The Belt and Road” based on network analysis

**DOI:** 10.1371/journal.pone.0221874

**Published:** 2019-09-06

**Authors:** Zhewen Liao, Zhongxing Wang, Kun Guo

**Affiliations:** 1 School of Economics and Management, University of Chinese Academy of Sciences, Beijing, PR China; 2 Key Laboratory of Big Data Mining and Knowledge Management, Chinese Academy of Sciences, Beijing,PR China; 3 Research Center on Fictitious Economy & Data Science, Chinese Academy of Sciences, Beijing, PR China; 4 Penghua Fund Management Co., Ltd, Shenzhen, PR China; University of Almeria, SPAIN

## Abstract

As of November 1, 2018, China's "One Belt and One Road" Initiative has involved 123 countries and promoted worldwide communication, cooperation and trade exchange. This paper constructs correlation networks of exchange rates among the countries along “The Belt and Road” and analyzes the risk contagion structure. It is found that when “The Belt and Road” initiative is initialized, countries in Eastern Europe occupy important positions in the network and play a vital role in the spreading of exchange rate risks; however, during the process of “The Belt and Road” initiative, the exchange rate risks are decentralized geographically, whereas they are centralized in countries that have in-depth communication and cooperation. The minimum Spanning Tree method is also proposed to investigate the structure of complex networks. It is found that the geographical link between exchange rate fluctuations and correlations among the countries has been strengthened while China has become an important node in the exchange rate network after the launch of “The Belt and Road” initiative. In addition, the influence and promotion of RMB has rapidly benefited from the initiative.

## Introduction

After the international financial crisis in 2008, the world economy has been recovering slowly. Global development is uneven, and countries still face big challenges to their development. In September 2013, China launched the initiative of jointly building “The Silk Road Economic Belt and the 21st-Century Maritime Silk Road” (i.e., “The Belt and Road” or “The B & R”), which has attracted attention from all over the world. “The B & R” Initiative is China's greatest international economic ambition, aiming at stimulating economic development in a vast region covering sub regions in Asia, Europe and Africa, which accounts for 64% of world population and 30% of world GDP, as well as striving for promoting orderly and free flow of economic factors, a highly efficient allocation of resources and the deep integration of markets[1]. As a systematic project, it includes policy coordination and facilitates connections, unimpeded trade, financial integration and people bonding[2]. In each of these areas, the exchange rate fluctuation is a vitally important factor that needs to be taken into account. “The B & R” connects countries by means of trade and economic cooperation. The increase in construction investment and capital flows has led to variations in currency exchange rates, which influences the trading and economic relationships the other way around. For China, its RMB currency has depreciated quickly in recent years. Meanwhile, most countries along “The B & R” have adopted floating exchange rate systems, and a volatile exchange rate can lead to serious risks for investment and project contracting abroad.

The exchange rate between two currencies (also known as a currency pair) is the rate at which one currency is exchanged for the other. In the classic framework of economic analysis, exchange rate volatility has a significant impact on the real economy [3–9]. For example, the depreciation of the exchange rate can effectively improve the terms of trade and promote exports. At the same time, it can cause price increases in imports and bring imported inflation. Furthermore, the depreciation of a currency can make the cost of foreign investment increase, bringing additional investment risks. Thus, the exchange rate is an important economic variable, especially when considering the trading cooperation between countries. Several research studies make an attempt to explain the dynamic structure of the exchange rate using historical data and use this structure to make future predictions [10]. The exchange rate is also regarded as the price of a country’s currency in relation to another currency. When the demand and supply of a currency changes, so does the exchange rate. Therefore, in most of the economic literature, exchange rates are generally thought to be determined by money supply, price levels, national income, interest rates, output and other related economic variables. A series of quantitative models that take these economic variables into consideration for exchange rate analysis have been proposed [11–16].

There are many factors that can influence the fluctuation of exchange rates. Correlations between exchange rates of different currency pairs display a complex pattern, which provides real-world material for network analysis. A complex network that can display the relationships between nodes in a network is first developed to study the topology structure of some real networks, such as the Internet, movie actor collaboration networks, citation networks, and so on [17–19]. In the field of finance, complex networks have been successfully applied to the stock market and the bond market to investigate the correlation structure of different assets and to obtain convincing conclusions [20–22]. However, the foreign currency market is also an important part of the financial market, but the relations between exchange rates have received scant research attention so far.

In this paper, the foreign currency market is regarded as a complex system that includes different exchange rates of currency pairs. The correlations between the exchange rate fluctuations of countries along “The B & R” route are used to construct the network. To be more intuitive and clearer, the Minimum Spanning Tree (MST) method is applied to analyze the influence structure. Thus, the key nodes and the key path of volatility risk contagion can be detected. This paper is organized as follows: the first section introduces the background to our research; the data are briefly introduced in section two; the techniques of network analysis and the results are discussed in section three; and finally, we end with a conclusion in section four.

## Data description

**Fig 1:**
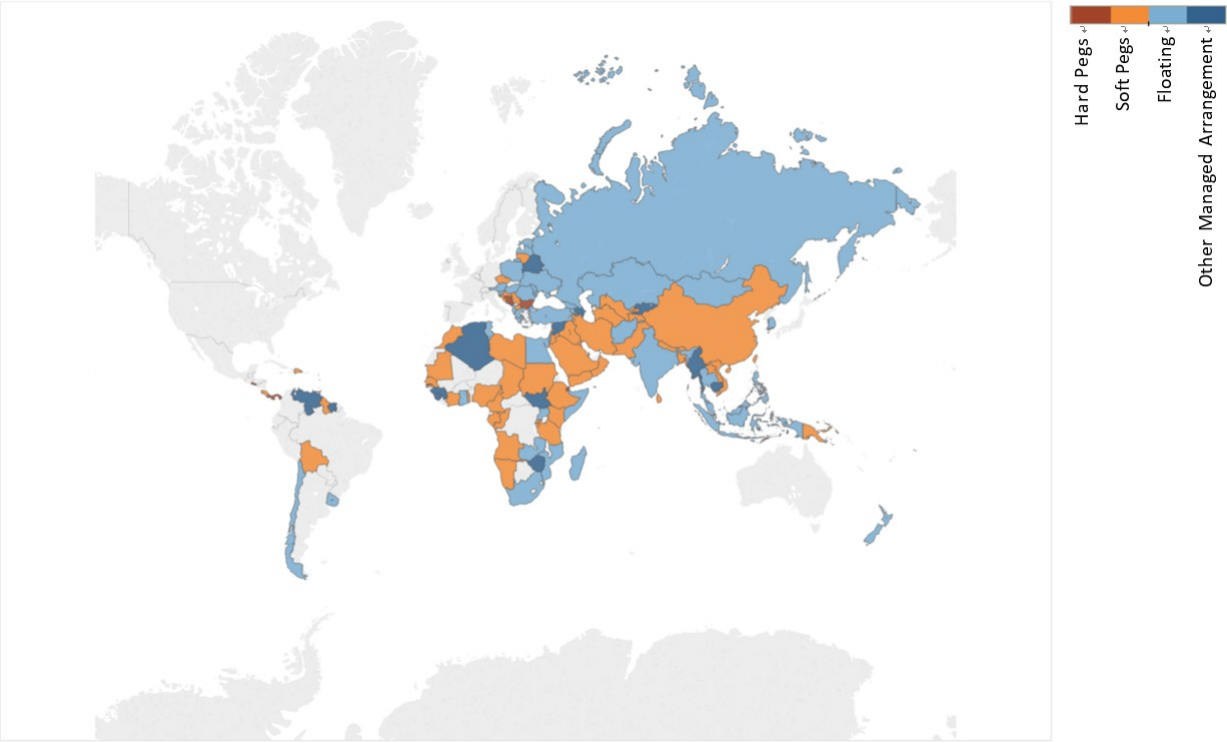
The exchange rate system of “The B & R” participants.

Up until November 1, 2018, over 123 countries, including China, have benefited from “The B & R” initiative, across Asia, Central and Eastern Europe, Southern Europe, Africa, the South Pacific, the Caribbean area and South America, which means that “The B & R” initiative covers over half of the planet. Our sample is composed of 126 daily settlement prices of exchange rates, including all the currencies in the countries that have benefited from “The B & R” initiative (the list of the countries is in S1 Table in Supporting Information). Three currencies with global influence are also included: the UK’s pound sterling, the EU’s euro and Japan’s yen. Given the limited data availability, the data period is not completely the same. All the exchange rates are compared with the US dollar. That is, they are the price of the currency denominated in US dollars. In this way, the analysis of the exchange rates is the same as the analysis of the price changes in a specific currency. The exchange rate regime and some statistics are shown in S1 Table and Fig 1.

The data preprocessing is as follows:

1. Empty the data filter process: In total, 10 countries that lack exchange rate data have been excluded from the sample network: Palestine, East Timor, Cape Verde, Djibouti, Madagascar, Morocco, Mozambique, Niue, Somalia and Chad. Next, the properties of the full sample network including all the remaining 115 countries and regions (including Japan, the UK, and the Eurozone) are identified.
2. Exclude countries that have not issued their own independent currencies: For the correlation network construction and analysis, countries in the Eurozone where the euro circulates, such as Greece, or currencies that were changed to the euro, such as that of Latvia, were omitted, and the countries in Africa that do not use their own currencies were also excluded. Nevertheless, the countries that have their currencies rate which pegged to the US dollar are counted in.
3. Select the time window:

1. The study period is from January 5, 2017, to November 1, 2018, because this period contains the largest amount of data that can be utilized (over 100 exchange rate data of different countries and regions per day during this time). When selecting this time window, it is necessary to abandon the Lithuanian data because it is empty. After the screening, a total of 102 countries and regions (including Japan, the UK, and the Eurozone) remained.
2. Second, a two-stage structure is used in our study. To identify the effects of “The B & R” initiative, the timeline should last as long as possible. The study period must contain the important date of September 2013, which is regarded as the point in time when “The B & R” initiative was first proposed. Therefore, the countries with a shorter available time period are deleted and we obtain a subsample that includes 58 countries and the Eurozone from January 1, 2010, to November 1, 2018. Given the launch time of the initiative in September 2013, the sample is divided into two parts: before the policy (abbreviated as “Bef,” from January 1^st^, 2010, to August 31^st^, 2013) and after the policy (abbreviated as “Aft,” from September 1^st^, 2013, to November 1^st^, 2018). The exchange rates that cannot cover this period are deleted, and 48 countries or areas are left to form the subsample, which still includes the majority of the important nodes identified in the previous full sample network. All the data were obtained from the Wind Co database.

### Analysis using the coefficient of variation

**Fig 2:**
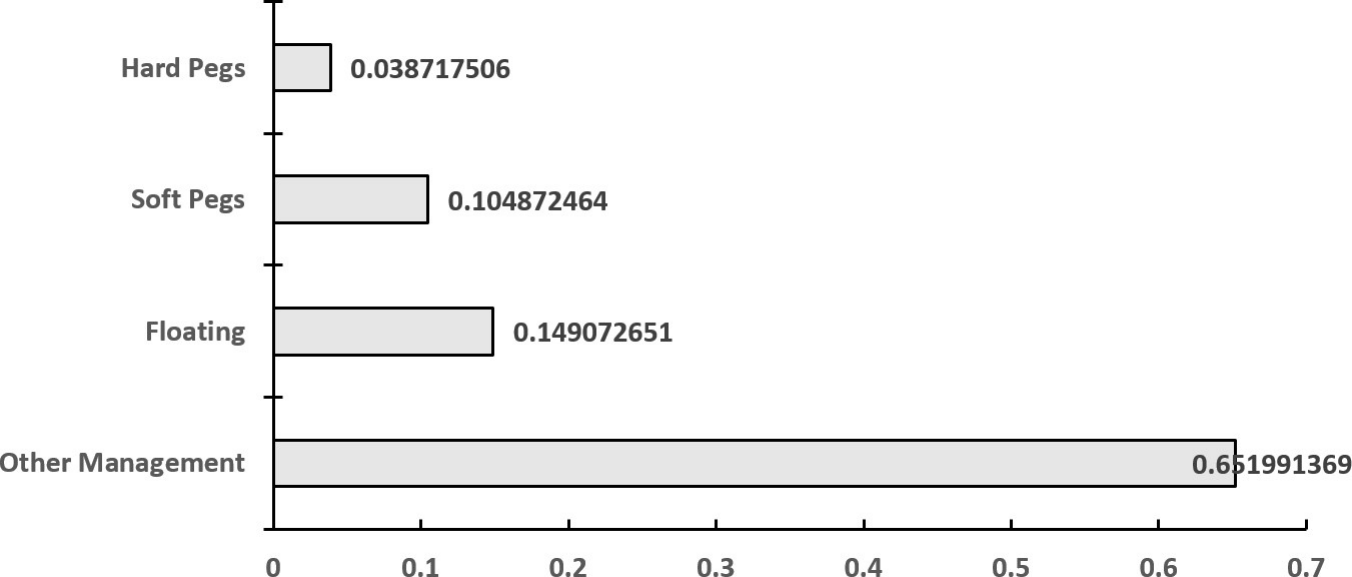
The average C.V. of different exchange rate systems of “The B & R” countries.

As shown in S1 Table, according to the IMF's official statistics, there are many kinds of exchange rate regimes among “The B & R” countries, most of which adopt a floating or a managed floating exchange rate system; the exceptions are Barbuda, Panama and a few other countries in the Caribbean, and in Southern Europe and East Africa, which have adopted a fixed exchange rate system. For this reason, the vast majority of countries usually incur higher risks and face increased volatility. To identify the relationship between exchange rate regimes and exchange rate volatility, as well as to eliminate the influence of the measurement dimension, which is defined by the standard deviation to the average value ratio, the coefficient of variation (C.V.) of each exchange rate is calculated to reflect the fluctuation risk of each country. Generally, as depicted in Fig 2, hard pegged exchange rate systems have the least volatility, followed by soft pegged exchange rate systems; other managed-arrangement exchange rate systems have the highest volatility, which is consistent with our intuition that the exchange rate volatility in fixed-exchange-rate countries is necessarily weaker than those in floating-rate countries.

**Fig 3:**
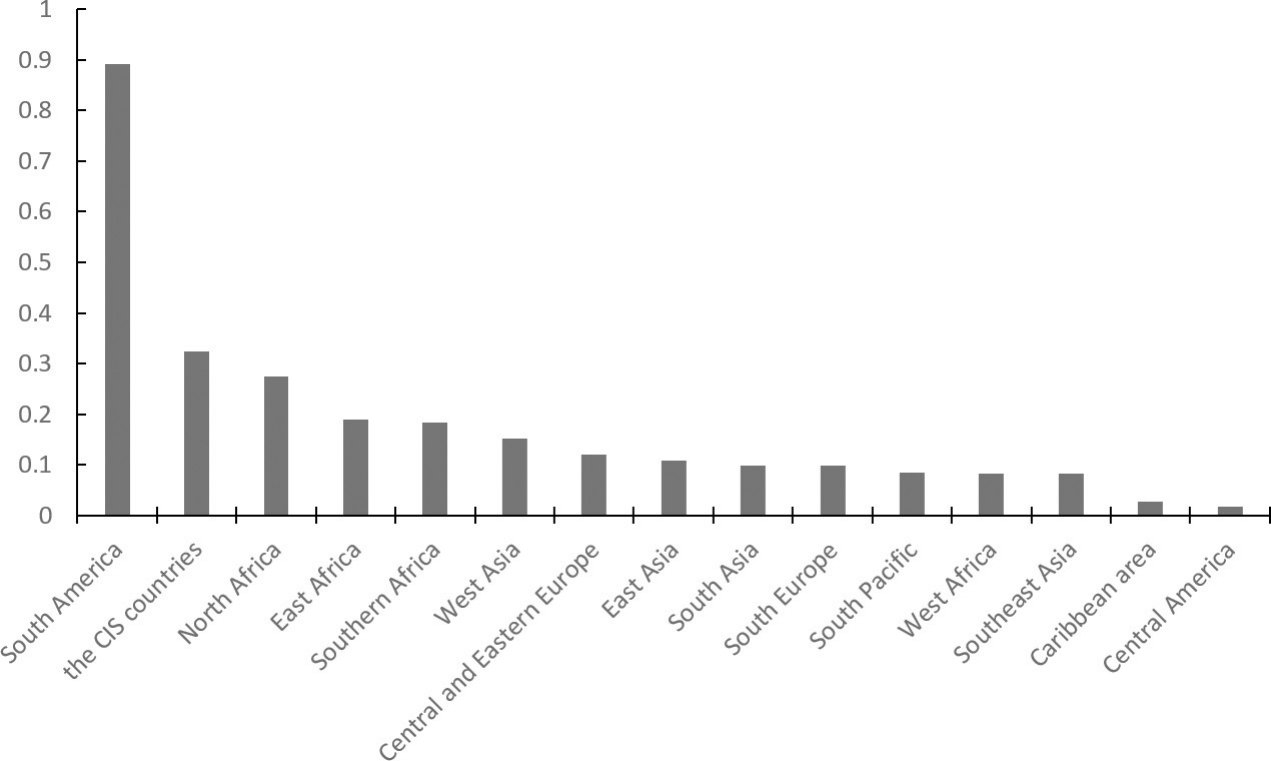
Regional distribution of the coefficients of variation.

When it comes to different countries, all “The B & R” countries involved have been divided into 15 geographical regions. According to the coefficient of variation, the coefficients of variation of Venezuela, South Sudan, Belarus, Syria, Sudan, and Uzbekistan are significantly larger than those of the other countries (as shown in S1 Table). From the perspective of regional distribution, as shown in Fig 3, the exchange rate volatility can be divided into five levels. The first level includes countries in South America with the highest risk of exchange rate volatility. Second, the Countries of the Commonwealth of Independent States (CIS) and North Africa are at a great risk of exchange rate volatility. The middle level includes East Africa, South Africa and West Asia. The fourth level includes Central and Eastern Europe, East Asia, South Asia, Southern Europe, the South Pacific, West Africa and Southeast Asia. The fifth level includes the Caribbean and Central America, with the least risk of exchange rate volatility. However, from a holistic point view, it has been concluded that fixed-exchange-rate countries are necessarily weaker than floating-rate countries, as shown in Fig 2.

**Fig 4:**
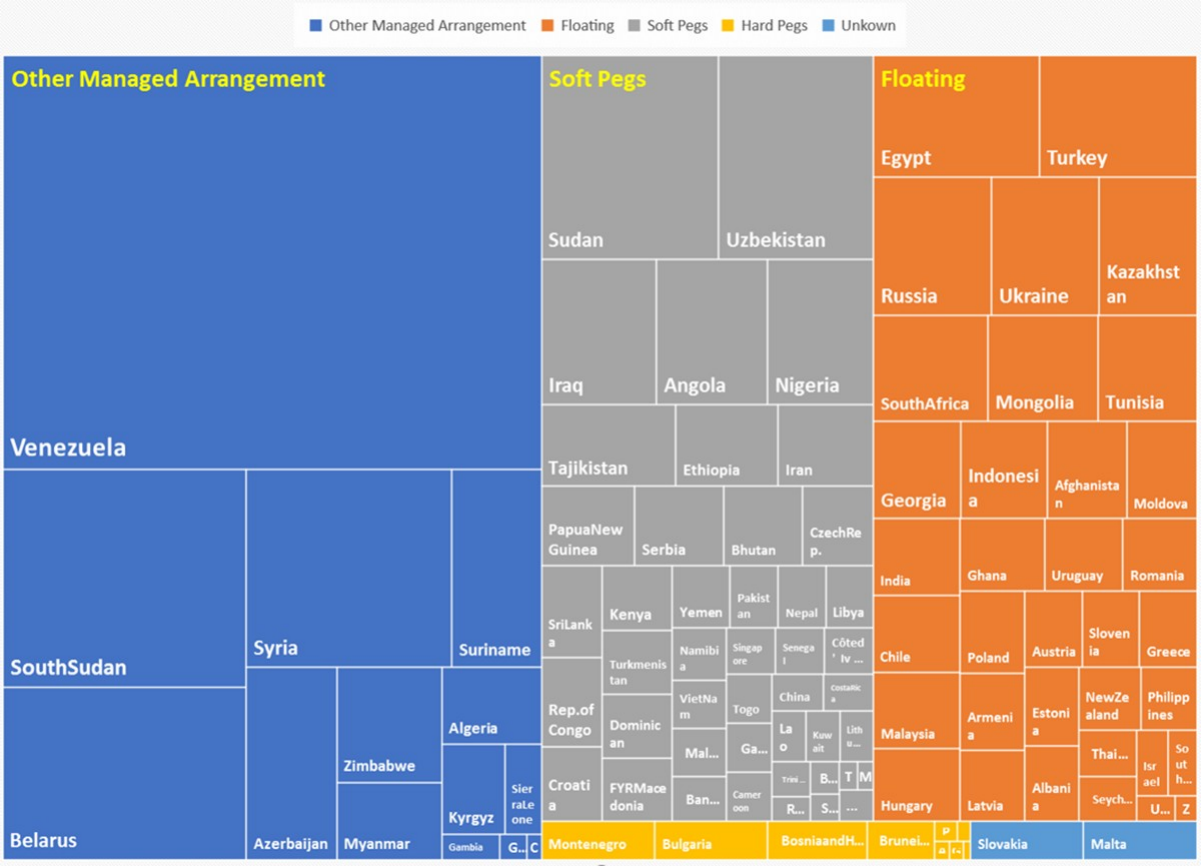
Country distribution by coefficient of variation and different exchange rate regimes.

In Fig 4, the area of the square named by the country represents the C.V. of the country, and it can be seen clearly that the relationship between a country’s exchange rate fluctuation and its exchange rate regime does not always correspond to the abovementioned viewpoint; it can therefore be easily seen that the soft peg systems also have diamond area that are larger than the floating system and other managed-arrangement diamonds area, as illustrated by Sudan and India, which makes it more difficult for foreign investment to identify and prevent foreign exchange risks.

### Financial market openness and exchange rate fluctuation

It is hardly to measure the openness of financial market in a direct way, the observers of capital flows as the proxy variable is widely used as the proxy variable in the research domain. There are two methodologies to measure the capital flow: (1) De jure, which has been categorized by Kose[23]; (2) De facto. De facto measures based on price differentials has been introduced by Cheung[24] and the other measuring methods which based on quantities, can be seen in the work done by Lane and Milesi-Ferretti[25].

The IMF’s Annual Report on exchange Arrangements and Exchange Restrictions (AREAER) is widely used in attempting to measure the openness of financial market. AREAER provides information on the extent and nature of the rules and regulations governing external account transactions for a wide cross-section of countries since 1967. A summary table is contained in AREAER that conveniently enumerates the presence of restrictions for the countries, providing the basis for researchers to come up with the dichotomous measure of or financial openness.

Based on the AREAER tabulation, with the time variable and measuring the strength and breadth of capital control considered, the KAOPEN Index method is constructed by Chinn and Ito[26].

Since the indicator not only take the control of the capital account into consideration, but also measures the influence of other factors on capital flow, considering the capital control or openness in a holistic view, and is widely used in the research domain[27].

**Fig 5:**
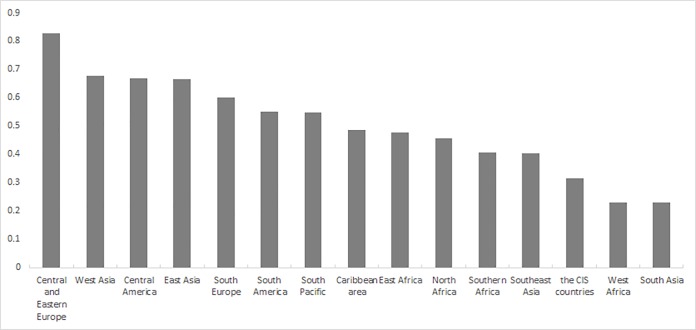
Regional distribution of the KAOPEN Index.

The KAOPEN Index data which has been updated to 2016 is used in our analysis. For better representation, 2016 data has been chosen for the whole period since the “B & R” initiation began. Like the C.V. analysis, as the Fig 5 depicted, the KAOPEN Index is a normalized indicator, which means that the differences between different regions are not as significant as the indicator like C.V. These regions can roughly be divided into three levels: higher than 0.7(high), 0.7–0.6(medium) and below 0.6(low). The first tier includes countries in Central and Eastern Europe, Central America and South Europe, which could be concluded that the Europe and Central America have relatively high financial market openness and less capital control. Second tier includes the South Pacific, West Asia, East Asia and South America, which have the relatively moderate financial market openness. The financial market openness of rest area in the “B & R” route. However, from a holistic point view, it could be referred that the relative developed regions like Europe and America have high financial market openness and less capital control. But compared with Fig 3, it can be found that the strict capital flow control could not stabilize the exchange rate. For example, the CIS countries have high exchange rate volatility while the financial market openness is low; on the contrary, Central America has the most stable Exchange rate with less capital control.

In Fig 4, the area of the square named by the country represents the C.V. of the country, and it can be seen clearly that the relationship between a country’s exchange rate fluctuation and its exchange rate regime does not always correspond to the abovementioned viewpoint; it can therefore be easily seen that the soft peg systems also have diamond area that are larger than the floating system and other managed-arrangement diamonds area, as illustrated by Sudan and India, which makes it more difficult for foreign investment to identify and prevent foreign exchange risks.

**Fig 6:**
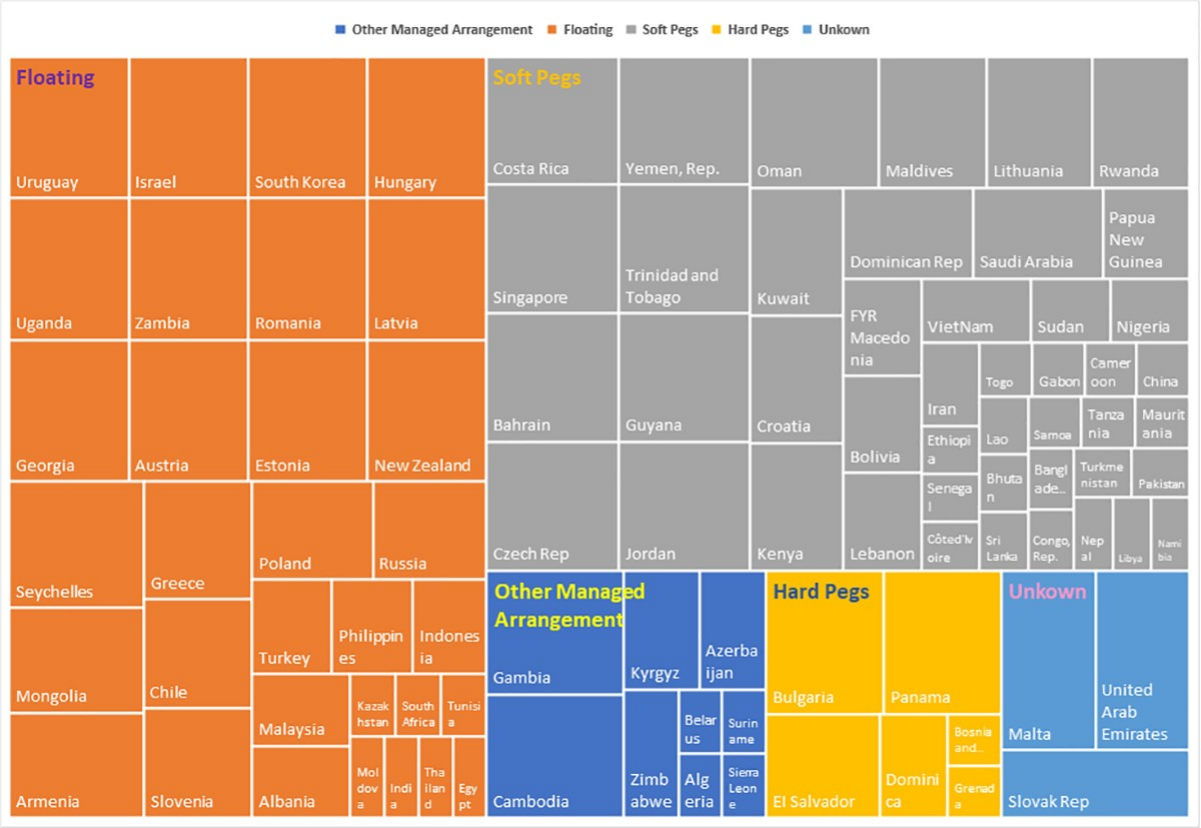
Country distribution by KAOPEN Index and different exchange rate regimes.

**Fig 7:**
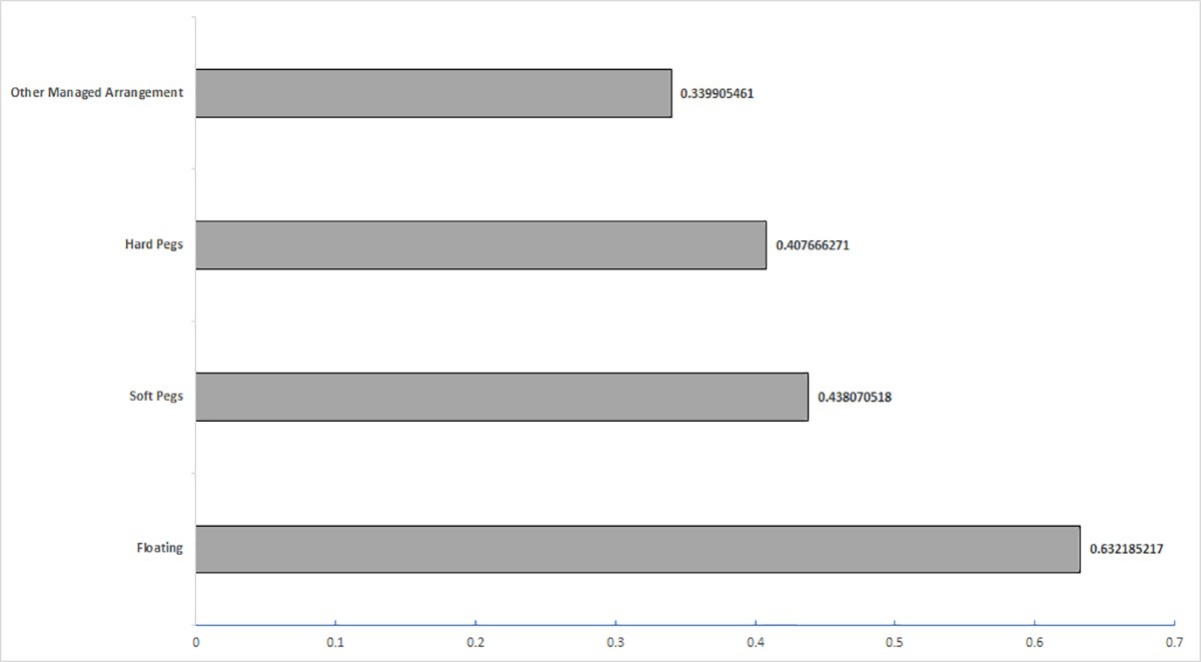
Country distribution by KAOPEN Index and different exchange rate regimes by bar chart.

Combined with Figs 6 and 7, it can be clearly seen the relationship between a country’s financial market openness and its exchange rate regime. The countries which have floating system have the highest financial market openness, follow by soft pegs system, and then the hard pegs, just as the economic intuition. It could be concluded that in the “B & R” route, the degree of freedom of exchange rate system usually has a positive correlation of the financial market openness.

In addition, the OECD’s FDI Regulatory Restrictiveness Index has also been introduced and used in the part named Influence on financial market openness from “The B & R Initiative”.

## Correlation network of the exchange rates of countries on “the Belt and Road” route

### Correlation network and minimum spanning tree network

A complex network consists of several nodes and edges linking them. The node is the basic element of a complex network, which is the abstract expression of an “individual” in the real world. The edge is an expression of the relationship between the elements and can be given weight according to the extent of the relationships. Here, *w*_*ij*_ represents the weight of the edge linking node *i* and node *j*, where *i*, *j = 1*,*2*,*3*,*…*,*n* and *n* is the amount of nodes in a certain network. For an undirected network,

$$w_{ij}=w_{ji}$$ (1)

We can also use the weighted degree to represent the importance of nodes, which is defined as:

$${dw}_{i}=\sum_{j\in v(i)}w_{ij}$$ (2)

where *v*_(*i*)_ is the set of nodes linking to node *i*. The larger the weighted degree, the stronger is the degree of correlation with other nodes and the more important the node is.

In this study, the original exchange rate data {*X*_*it*_,i = 1,2,3,…,n;t = 1,2,…,T} have to be standardized, due to significant differences at the numerical level, that is:

$$Y_{it}=\frac{X_{it}-\bar{X_{i}}}{std(X_{i})}$$ (3)

We use the exchange rates of countries on “The B & R” route as the network nodes and construct the network using correlation coefficient *ρ*_*ij*_ as the edge weight.

$$w_{ij}=\rho_{ij}=<Y_{it}∙Y_{jt}>$$ (4)

where <…> indicates a time-average over the T data points for each time series.

To detect the correlation structure of the exchange rate network, we apply the minimum spanning tree (MST) [28] method that has been previously applied to exchange rates [29]. This method selects the indices with the closest interactions among all the indices and generates a visual presentation of the relationship with n-1 edges in the tree. When using the MST, the relatively unimportant edges are discarded and there is only one route between any two nodes, which means that the complex network constructed by the MST shows more concise and clearer exchange rate volatility relationships between “The B & R” countries and that it is easier to discern the key countries in the exchange rate volatility complex network.

To construct the MST, we first need to convert the correlation coefficient into a “distance” coefficient. Following these references [20, 29], we use nonlinear mapping

$$d_{ij}=\sqrt{2(1-\rho_{ij})}$$ (5)

to get the distance *d*_*ij*_ of two nodes from the correlation coefficient *ρ*_*ij*_. Since −1<*ρ*_*ij*_<1, we have 0<*d*_*ij*_<2. The Kruskal algorithm [30] is used in this paper to construct the MST.

### A full-sample analysis

**Fig 8:**
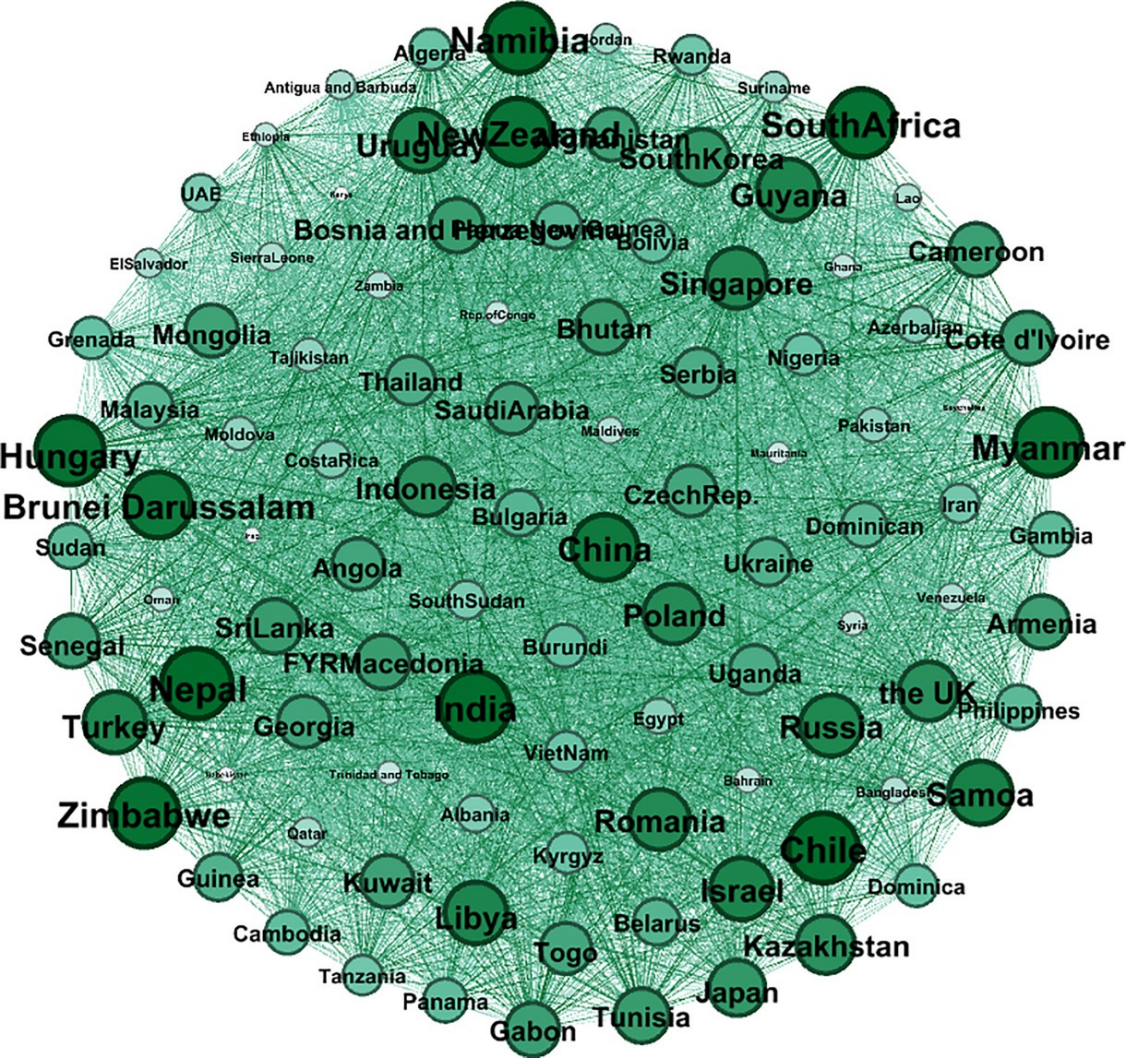
The correlation network of the full sample.

Fig 8 shows the correlation network of the full sample. The intensity of the color depends on the weight of the edge, the darkness of the edges is related to the weight, and the size of the node refers to the weighted degree. The larger the node, the more closely the country’s currency fluctuates with neighbor nodes. The exchange rates of different currencies in “The B & R” are highly correlated. With the strengthening of trade and economy, foreign currency markets in this area behaved more and more like a whole. For a network with *n* nodes, there will be *n(n-1)/2* correlation coefficients according to the definition mentioned before. Clearly, a complicated relationship is shown by the network, which indicates that Nepal, India, Chile, Namibia, Zimbabwe, South Africa, Hungary, Myanmar, New Zealand, Brunei Darussalam, China, Samoa, Uruguay, Israel, Guyana, Libya, Turkey, Russia, Singapore, and Romania are the top 20 nodes, mainly distributed across South Asia, Southeast Asia, Southern Africa and Eastern Europe, and that it has the characteristics of a trade partner cluster and a geographical regional cluster. For example, South Africa has strong links with Namibia and Cameroon and Cote d'Ivoire, which shows that geographical proximity results in a strong exchange rate volatility transmission network, and the strong edge between China and the UK, which are over 8000 kilometers apart in terms of their geographical distance, demonstrates the power of international trade: China is the UK's third largest trading partner after the EU and the US and the UK's largest source of investment. As long as the number of nodes is given, the number of the edges is fixed, and hence, it is extremely difficult to deduce which correlations are the most important for controlling the overall structure of the system by observing such a complex network with 5151 edges. To get a clearer and more concise transmission network of exchange rate volatility, the MST algorithm is used.

**Fig 9:**
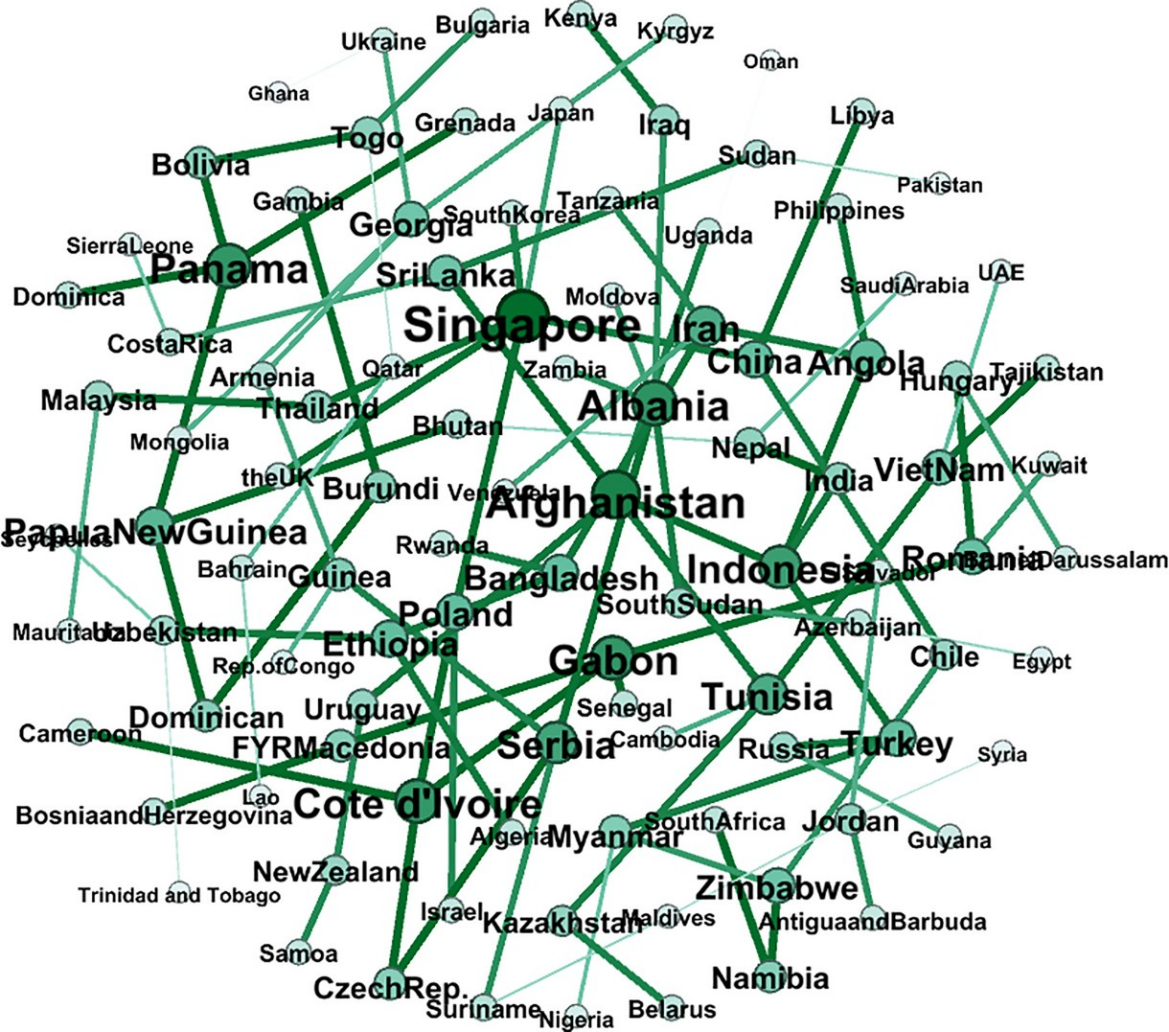
The MST network of the full sample.

The MST network that we construct from the full sample network is shown in Fig 9. Broadly speaking, each node is linked to the nodes that represent the exchange rates to which the node is most closely correlated. Thus, the clustering structure shows that these currencies move together consistently in the research period. Remarkably, there is a geographical link between the exchange rates. China is linked to Kazakhstan, Sri Lanka, Kyrgyzstan and Bangladesh. These countries are also geographically adjacent. A similar situation can be observed in the case of the Southern Asia nodes (Indonesia, Malaysia, Thailand, Singapore, etc.) and Eastern European nodes (Czech Republic, Serbia, Croatia, Macedonia, Bosnia, Bulgaria, Hungary, etc.) The transactions between neighboring countries are usually more active, so that the currency prices fluctuate in a similar way.

Furthermore, the key nodes in the exchange rate network and the MST are basically hard or soft pegged exchange rate countries; Gabon can be taken as an example because its Central African Franc currency has an exchange rate that is fixed not only to the main currencies like the Euro or the West African Franc but also to the Central African Franc used by Gabon’s neighbors. As long as the currency fluctuates, the same amplitude of fluctuation is transmitted rapidly in the network due to the fixed exchange rate.

### A comparative analysis of two stages

**Fig 10:**
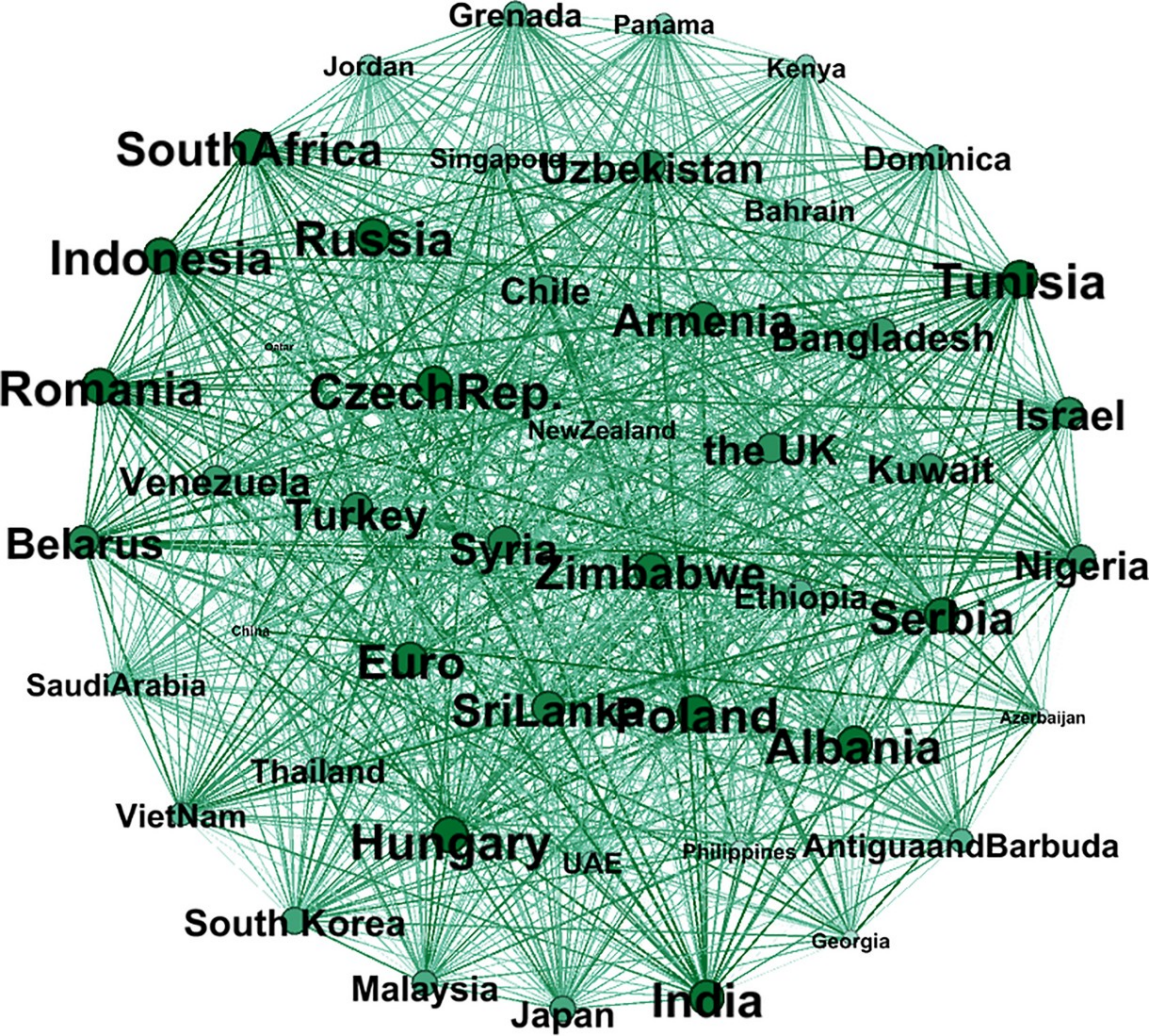
The correlation network composed of the subsample before “The B & R” initiative.

In this section, the effect of “The B & R” initiative is examined in two subperiods. As can be seen from the correlation network in Fig 10, the top ten countries with the largest weighted degree are the following: Czech Republic, Hungary, Tunisia, Poland, Albania, Russia, Romania, India, Indonesia and Serbia. In addition, the 11^th^ is Euro, with a 91.1% weighted degree compared to 1^st^ place: Czech Republic. The details are shown in S1 Table in Supporting Information. This implies that the fluctuation of currencies in these countries is very important among all the countries involved in “The B & R” initiative. The enterprises and the government of China should see the Eastern European countries as occupying a vital position in order to avoid exchange rate risks.

At the same time, the correlation between the dollar-denominated RMB exchange rate and other countries is weak, making China a relatively isolated node and occupying the penultimate position. Despite the gradual use of a managed floating exchange rate system in China, the volatility of RMB depends on the US dollar to a considerable extent, while the exchange rates of other floating exchange rate countries are not pegged to the US dollar.

**Fig 11:**
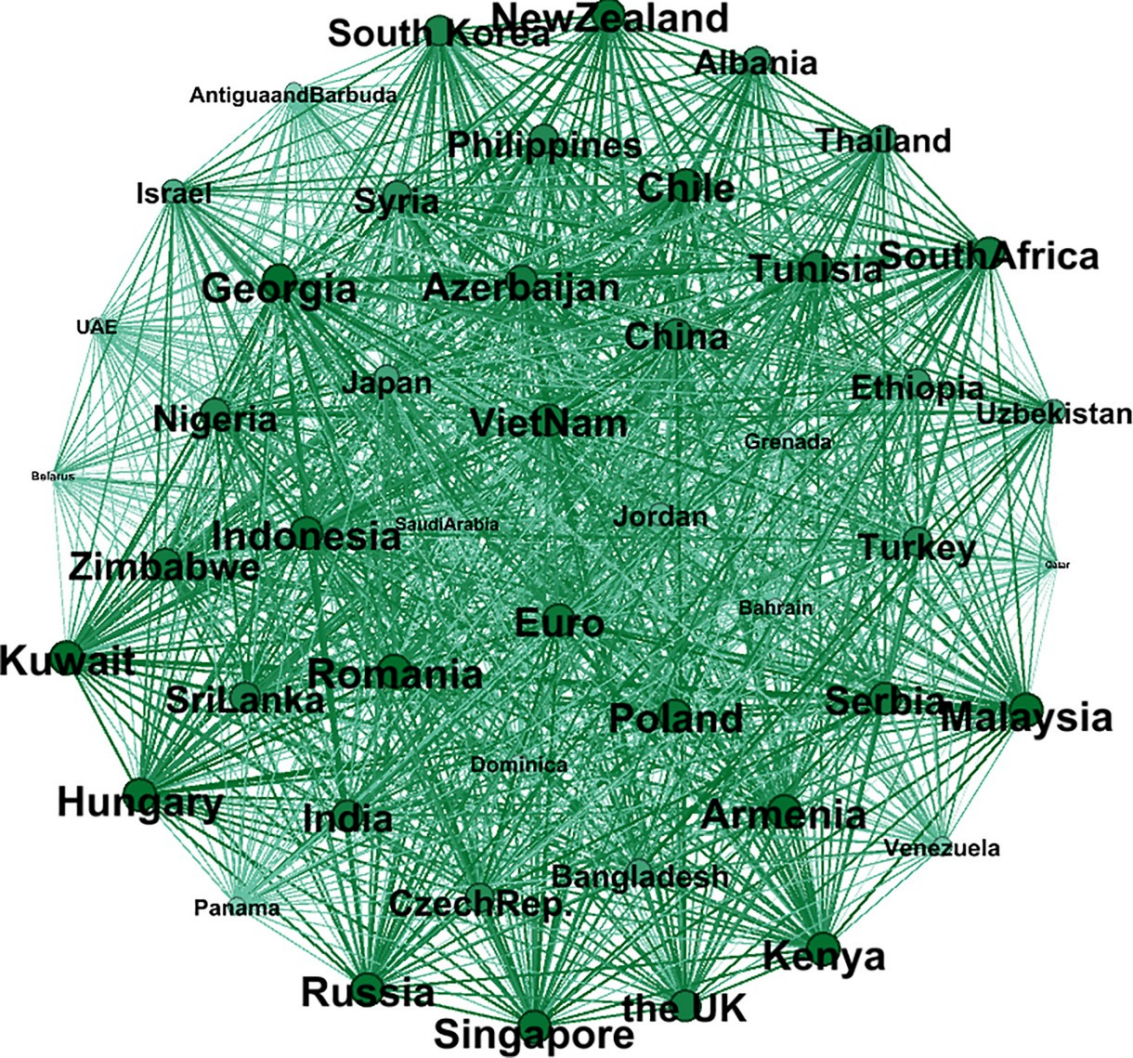
The correlation network composed of the subsample after “The B & R” initiative.

**Fig 12:**
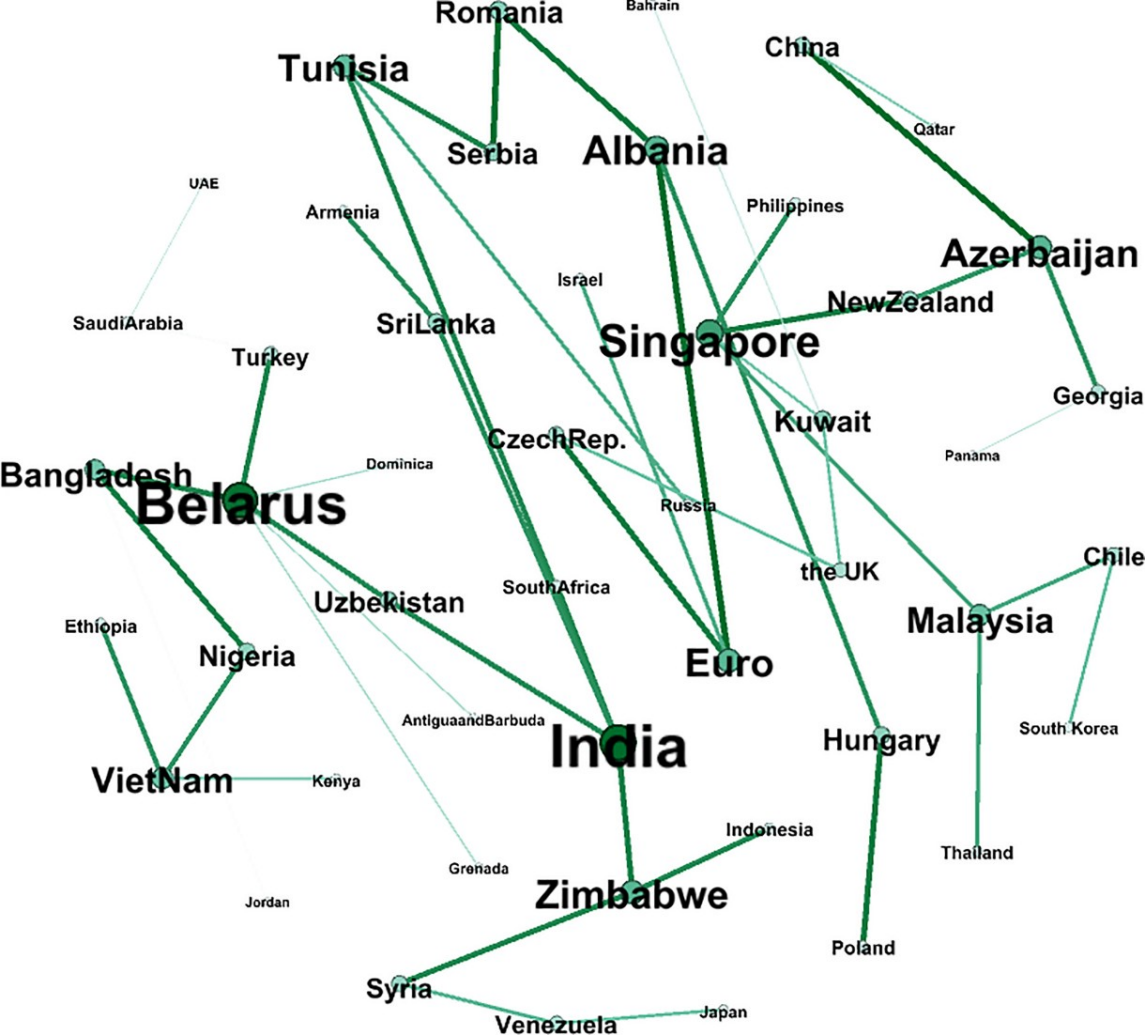
The MST network composed of the subsample before “The B & R” initiative.

**Fig 13:**
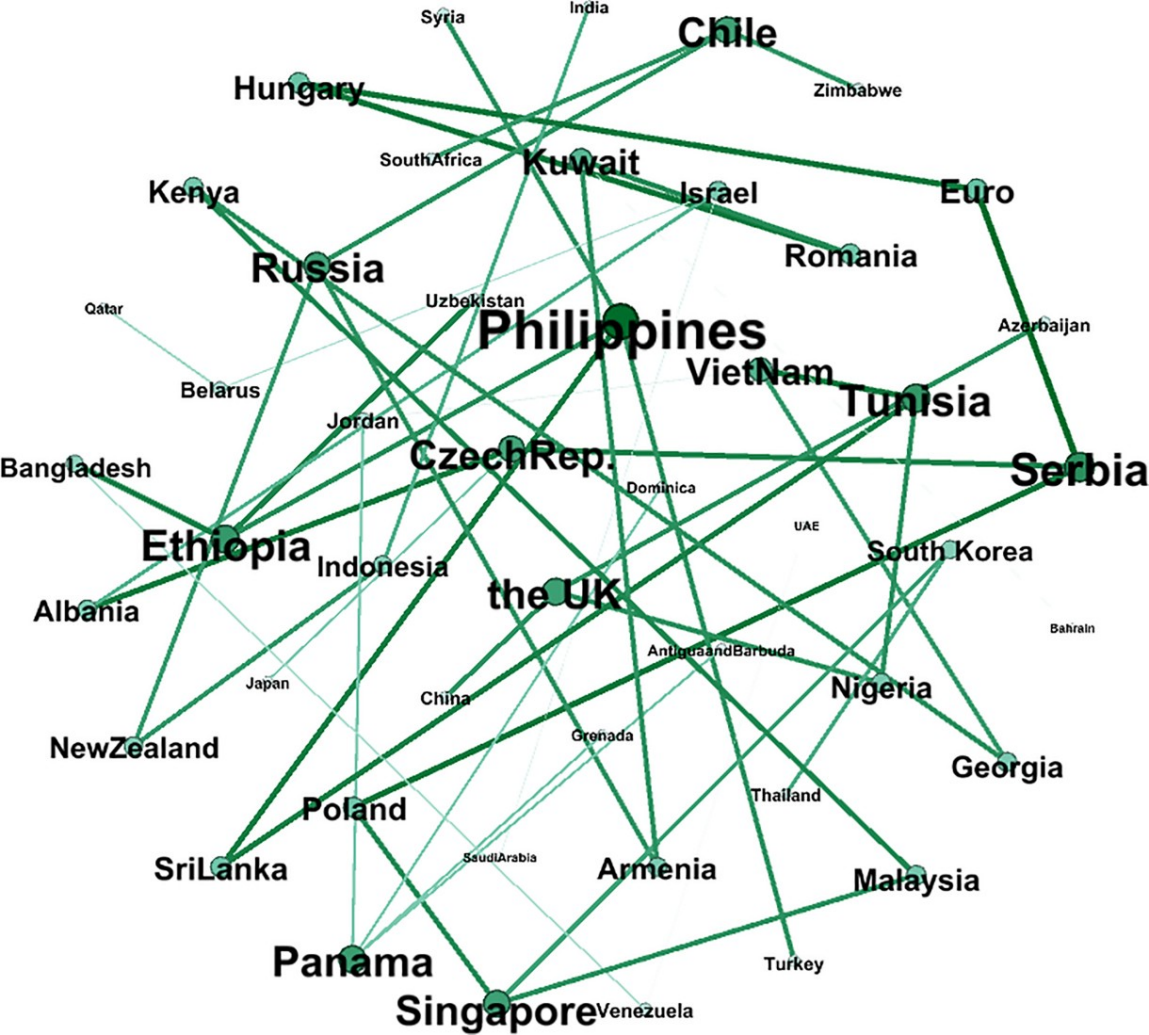
The MST network composed of the subsample after “The B & R” initiative.

Figs 10 and 11 show a comparison of the exchange rate networks before and after “The B & R” launch. In addition, Figs 12 and 13 depict in the MST way, and the MST in Fig 12 shows a dissimilar geographical distribution compared to Fig 9. The top ten countries with the largest weighted degree are Kuwait, Romania, Armenia, Malaysia, Georgia, Kenya, Hungary, Russia, Singapore and New Zealand. In addition, the Euro dropped 3 places to the 14^th^ spot but still has a 91.9% weighted degree compared to Kuwait, which is in 1^st^ place. The details are shown in S1 Table in Supporting Information. This means that given the processes of “The B & R” initiative, the key nodes in the network have been changed and decentralized to a wider geographical area, like East Africa, Southeast Asia, South Asia and Europe, instead of only Eastern Europe. That is, the volatility of the exchange rate faced by the enterprises and the government of China has been diversified, and so they should pay attention to more of the countries involved in “The B & R” initiative.

To measure the degree of correlation of the whole network, we calculate the weighted average of every node’s weighted degree in the network. Before “The B & R” initiative was launched, the average weighted degree was 15.589, and it increased to 35.439 after the initiative launch. This reflects the strengthening of the correlation between the currencies of the countries along the route and of the strengthening of links in trading and economic cooperation. On the other hand, it implies that the exchange rate risk will diffuse rapidly among countries along the route.

It is also clear that the weighted degree of China has improved after “The B & R” initiative was put forward, from -20.57 to 44.87. This suggests that this Chinese initiative has significantly improved the status of RMB internationalization. Taking note of the nodes with the largest weighted degree, Singapore increased dramatically according to our research. Despite being a small country with a population of less than 6 million, Singapore plays an important role as a hub on the maritime Silk Road and achieved a significant increase in its strategic position.

Table 1 shows the top twenty countries that exhibit the greatest changes in their weighted degree between Bef and Aft. Surprisingly, China dominates this ranking. As the initiator of “The B & R” initiative, China occupied the top position with a 65.44 promotion of weighted degree, that is, the influence of the Chinese RMB has improved a lot. The table clearly shows that the countries of Southeast Asia, South Asia, Eastern Europe and East Africa obtain most of the positions, but compared to their prominence in the other networks above, the Eastern European countries do not stand out in Table 1, which means that the influence of these countries’ currencies did not increase with “The B & R” initiative.

**Table 1:** The change in weighted degree between Bef and Aft.

Ranking	Country	Weighted degree change	Ranking	Country	Weighted degree change
1	China	**65.44642**	11	Chile	**35.71127**
2	Azerbaijan	**63.79049**	12	South Korea	**28.37031**
3	Georgia	**63.45171**	13	Armenia	**26.24802**
4	New Zealand	**54.72056**	14	Thailand	**24.94593**
5	Kenya	**54.09654**	15	Ethiopia	**24.90396**
6	Singapore	**52.62191**	16	the UK	**24.5254**
7	Philippines	**50.78511**	17	Nigeria	**22.8467**
8	Malaysia	**40.98127**	18	Jordan	**22.24446**
9	Vietnam	**40.524**	19	Romania	**21.41102**
10	Kuwait	**36.8171**	20	Indonesia	**19.83508**

### Influence on financial market openness from “The B & R Initiative”

The influence on financial market openness from “The B & R Initiative” also has been considered because there might be some connection between the financial openness and the foreign exchange market.

**Fig 14:**
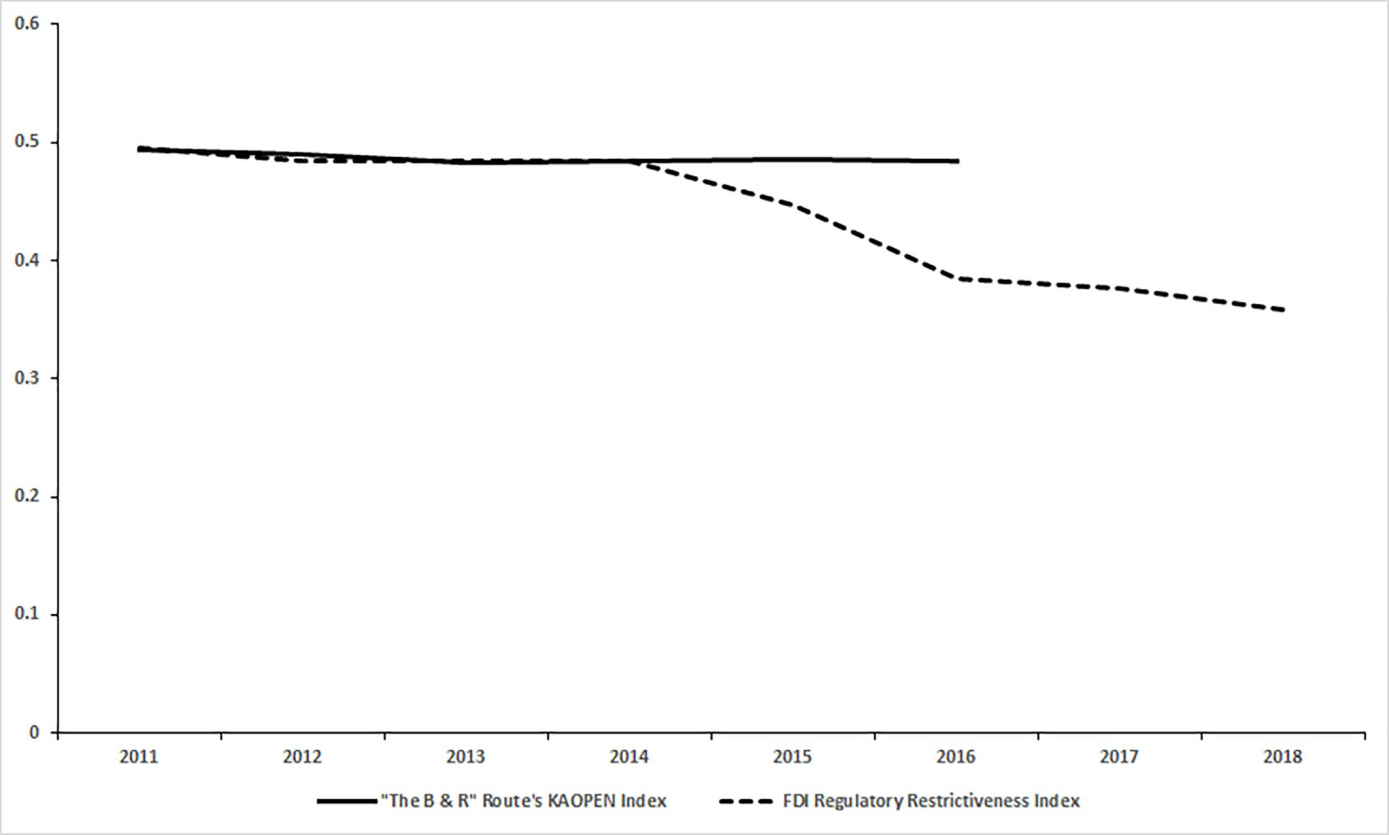
The trend of the participants of the B & R route and China.

The Fig 14 has depicted the trend of the financial market openness of the countries along “The B & R Initiative” route indicated by the average KAOPEN Index of all the countries along “The B & R Initiative” route which have available data in the period from 2010 to 2016. Beyond our intuition, the trend of the KAOPEN Index is downward slightly, not far which means that “The B & R Initiative” did not make much significant change, although the relevance within the system of the countries along “The B & R” route is strengthened compared with the period before “The B & R Initiative”. For the purpose of verifying this conclusion, the OECD’s FDI Regulatory Restrictiveness Index has also been used. The FDI Regulatory Restrictiveness Index measures statutory restrictions on foreign direct investment across 22 economic sectors[31]. OECD has a limited number of participating countries which could not cover all “The B & R” route, so only data of several systematic important countries could be found. Not surprisingly that the change of The FDI Regulatory Restrictiveness Index for most countries is not significant, it is to be speculated that the reason might be the financial market openness and the restriction of FDI for a country are likely to be endogenous, decided by the government especially the country’s central bank who are more likely to achieve some long-term goals, which could not be easily effected by the foreigner’s initiative in a short period. In addition, as shown in Fig 14, the conclusion is that “The B & R Initiative” makes China a more open country with less Financial restrictions for the foreigner, and at this situation, China’s currency, as known as RMB, would plays a more important role in the economy of the world.

## Conclusions

There are over 123 countries distributed along “The B & R” route, in Asia, Europe, Africa, South America and Oceania. Most of the countries have adopted floating or managed-floating exchange rate regimes, which correlate closely to those of other countries and make them more vulnerable.

Despite the increasingly prominent role of “The B & R” initiative, there are widespread problems, such as insufficient foreign exchange reserves and the fragile financial systems of these countries along “The B & R” route, as is partly shown in the analysis of the exchange rate fluctuations above. In the event of an external financial shock or something that causes an exchange rate fluctuation, international trade and economic cooperation at both the national and enterprise level would be greatly affected. And from our research it could also be concluded that in the “B & R” route, the degree of freedom of exchange rate system mainly has a positive correlation of the financial market openness.

In this paper, we construct an innovative correlation complex network and an MST of exchange currency rates along “The B & R” route. The analysis based on complex networks and statistical analysis shows that the correlations among countries’ exchange rates are gradually being strengthened, while the correlation between the dollar-denominated exchange rate of the RMB and other countries that have adopted the free-floating system is relatively weak. This finding suggests that enterprises in China face more exchange rate fluctuation risks in the process of implementing “The B & R” initiative. At the same time, we can see that among the countries along the route, the currencies of Kuwait, Romania, Armenia, Malaysia, Georgia, Kenya, Hungary, Russia, Singapore and New Zealand and the euro play an important role in the correlation network. These countries’ and regions’ exchange rates have been remarkably volatile in recent years, leading to volatility in the whole network along the route. Once the exchange rate of the key node countries fluctuates, it quickly spreads to other relevant countries.

There is also strong evidence that “The B & R” initiative has changed the world of currencies. Using the two-stage complex network and the MST network construction and comparison, it can be concluded that the influence of a currency always changes along with the changes in the international situation. “The B & R” initiative, from September 1^st^, 2013, to November 1^st^, 2018, benefited the weighted degree of China, which improved to 65.44, followed by Azerbaijan, Georgia, New Zealand, Kenya, Singapore, Philippines, Malaysia, Vietnam, Kuwait, Chile, South Korea, Armenia, Thailand, Ethiopia, the UK, Nigeria, Jordan, Romania, and Indonesia. Moreover, the average weighted degree increased from 15.59 to 35.35, which means that with the deepening of communication and cooperation among different countries “The B & R” initiative makes the currencies from these countries involved become more embedded in the network of exchange rate fluctuations, and undoubtedly, there is still an enormous space for RMB receipts and payments in trade and investment along “The B & R” route. As for China, thanks to “The B & R Initiative”, compared to the average level of the change of the openness of the financial market, China’s financial market openness had a significant promotion, with less financial restrictions for the foreigner. RMB might become the most commonly used infrastructure investment and financing currency; RMB internationalization can achieve considerable gains from the financial business carried out along the route and promotion of financial market openness brought by “The B & R Initiative”.

Based on the conclusions above, corresponding policy recommendations can be put forward.

First, it is recommended to strengthen the monitoring and early warning systems of exchange rate fluctuations at the national level, especially for those countries that play an important role in our study. Combined with the long-run prediction of exchange risks by using macroeconomic variables, this could reliably support investment decision making and risk management.

Second, it is also important to increase the number of overseas financial branches to facilitate the local currency settlement business along “The B & R” route. At the same time, financial institutions should be encouraged to develop appropriate exchange rate hedging products to help relevant enterprises hedge the local currency risk to some extent.

At the same time, a signed currency swap agreement with countries along the road could promote trade in the RMB settlement mechanism. The RMB still cannot become a settlement currency in “The B & R” countries, as settlements in local currency often face double exchange rate risks. The signing of swap agreements and the settlement of RMB can reduce exchange rate risks in trade and foreign investment and simultaneously promote RMB internationalization.

Finally, it might be instrumental to create “The B & R” Bank that could perform a function similar to “the IMF plus the World Bank plus an investment bank and a commercial bank,” which would bring “The B & R” countries closer, especially in the commercial and financial domain.

If “The B & R” unified currency zone could be built similarly to the establishment of the Eurozone, based on “The B & R” economic integration, then the internationalization of the RMB would be indisputably faster, and the Chinese RMB would act as a stabilizer in the fluctuation of the currency exchange rates of “The B & R” countries.

## Supporting information

S1 Table The statistical description of exchange rate data about “The B & R” participants. (DOCX)

S2 Table The statistical description of exchange rate correlation network about “The B & R” participants before “The B & R” Initiative. (DOCX)

S3 Table The statistical description of exchange rate correlation network about “The B & R” participants after “The B & R” Initiative. (DOCX)
