# Supplementary material for: The dynamic evolution of the characteristics of exchange rate risks in countries along “The Belt and Road” based on network analysis
Source: PLoS One. 2019 Sep 6;14(9):e0221874. doi: 10.1371/journal.pone.0221874 (PMC6730902; doi:10.1371/journal.pone.0221874)
Supplement: S1 Table — (DOCX) [file pone.0221874.s001.docx]

S1 Table. The statistical description of exchange rate data about “The B & R” participants

| **Region** | **Country** | **Max** | **Min** | **Mean** | **Std Error** | **C.V.** | **KAOPEN** | **Regime** |
| --- | --- | --- | --- | --- | --- | --- | --- | --- |
| **the Commonwealth of the Independent States** | Armenia | 527.20 | 357.98 | 431.95 | 45.66 | 0.11 | 0.83 | Floating |
|  | Azerbaijan | 1.92 | 0.78 | 1.10 | 0.40 | 0.37 | 0.47 | Other |
|  | Belarus | 22069.00 | 1.85 | 6904.18 | 6091.12 | 0.88 | 0.17 | Other |
|  | Moldova | 20.99 | 12.63 | 17.26 | 2.48 | 0.14 | 0.17 | Floating |
|  | Russia | 83.31 | 27.29 | 45.17 | 15.48 | 0.34 | 0.66 | Floating |
|  | Tajikistan | 9.43 | 4.77 | 7.10 | 1.63 | 0.23 | 0.00 | Hard Pegs |
|  | Uzbekistan | 8236.87 | 1513.60 | 3061.62 | 2036.09 | 0.67 | 0.00 | Hard Pegs |
|  | Turkmenistan | 3.50 | 2.85 | 3.28 | 0.31 | 0.09 | 0.17 | Hard Pegs |
|  | Kazakhstan | 383.91 | 152.72 | 271.73 | 77.00 | 0.28 | 0.17 | Floating |
|  | Kyrgyz | 75.90 | 48.48 | 63.94 | 7.64 | 0.12 | 0.53 | Other |
| **South Asia** | Afghanistan | 118.88 | 73.30 | 95.41 | 15.51 | 0.16 | 0.00 | Floating |
|  | Bangladesh | 84.44 | 69.15 | 77.89 | 3.97 | 0.05 | 0.17 | Hard Pegs |
|  | Bhutan | 74.69 | 43.94 | 60.28 | 7.93 | 0.13 | 0.17 | Hard Pegs |
|  | India | 74.33 | 43.90 | 58.66 | 8.27 | 0.14 | 0.17 | Floating |
|  | Maldives | 15.64 | 12.61 | 15.10 | 0.81 | 0.05 | 0.83 | Hard Pegs |
|  | Nepal | 119.03 | 84.96 | 102.18 | 6.36 | 0.06 | 0.17 | Hard Pegs |
|  | Pakistan | 133.89 | 96.21 | 106.18 | 6.63 | 0.06 | 0.17 | Hard Pegs |
|  | Sri Lanka | 175.30 | 109.25 | 133.33 | 15.71 | 0.12 | 0.17 | Hard Pegs |
| **West Asia** | Bahrain | 0.39 | 0.36 | 0.38 | 0.00 | 0.01 | 1.00 | Hard Pegs |
|  | Georgia | 2.78 | 1.61 | 2.05 | 0.37 | 0.18 | 1.00 | Floating |
|  | Iran | 44120.00 | 24725.00 | 31206.76 | 5079.58 | 0.16 | 0.28 | Hard Pegs |
|  | Iraq | 1300.50 | 598.50 | 848.08 | 297.17 | 0.35 | 0.00 | Hard Pegs |
|  | Jordan | 0.72 | 0.70 | 0.71 | 0.00 | 0.00 | 1.00 | Hard Pegs |
|  | Kuwait | 0.31 | 0.27 | 0.29 | 0.01 | 0.04 | 0.70 | Hard Pegs |
|  | Lebanon | 1501.00 | 1501.00 | 1501.00 | 0.00 | 0.00 | 0.45 | Hard Pegs |
|  | Oman | 0.38 | 0.38 | 0.38 | 0.00 | 0.00 | 1.00 | Hard Pegs |
|  | Saudi Arabia | 3.76 | 3.74 | 3.75 | 0.00 | 0.00 | 0.70 | Hard Pegs |
|  | Syria | 517.56 | 45.40 | 210.60 | 179.78 | 0.85 | 0.00 | Other |
|  | Turkey | 6.94 | 1.39 | 2.54 | 1.03 | 0.40 | 0.45 | Floating |
|  | Yemen | 250.25 | 213.80 | 228.23 | 17.23 | 0.08 | 1.00 | Hard Pegs |
|  | UAE | 3.68 | 3.67 | 3.67 | 0.00 | 0.00 | 1.00 | Unkown |
|  | Israel | 4.08 | 3.36 | 3.70 | 0.16 | 0.04 | 1.00 | Floating |
| **East Asia** | China | 6.97 | 6.09 | 6.45 | 0.26 | 0.04 | 0.17 | Hard Pegs |
|  | South Korea | 1253.20 | 1008.85 | 1117.05 | 44.29 | 0.04 | 1.00 | Floating |
|  | Mongolia | 2513.13 | 1141.38 | 1904.85 | 470.09 | 0.25 | 0.83 | Floating |
| **Southeast Asia** | Brunei Darussalam | 1.52 | 1.19 | 1.33 | 0.07 | 0.05 | NA | Soft Pegs |
|  | Cambodia | 4122.00 | 3980.00 | 4047.86 | 32.67 | 0.01 | 1.00 | Other |
|  | Indonesia | 15261.50 | 8465.00 | 11478.47 | 2046.26 | 0.18 | 0.42 | Floating |
|  | Lao | 8639.75 | 7616.25 | 8008.72 | 294.70 | 0.04 | 0.17 | Hard Pegs |
|  | Malaysia | 4.50 | 2.94 | 3.56 | 0.50 | 0.14 | 0.42 | Floating |
|  | Myanmar | 1597.00 | 850.00 | 1148.44 | 196.72 | 0.17 | 0.00 | Other |
|  | Philippines | 54.35 | 40.57 | 45.83 | 3.44 | 0.08 | 0.45 | Floating |
|  | Singapore | 1.45 | 1.20 | 1.32 | 0.06 | 0.05 | 1.00 | Hard Pegs |
|  | Thailand | 36.59 | 28.66 | 32.51 | 1.82 | 0.06 | 0.17 | Floating |
|  | Viet Nam | 22727.00 | 17941.00 | 21119.30 | 1154.14 | 0.05 | 0.42 | Hard Pegs |
| **East Africa** | South Sudan | 13.89 | 0.30 | 4.73 | 5.28 | 1.12 | NA | Other |
|  | Ethiopia | 27.72 | 12.67 | 19.67 | 3.47 | 0.18 | 0.17 | Hard Pegs |
|  | Seychelles | 15.03 | 11.62 | 13.11 | 0.71 | 0.05 | 1.00 | Floating |
|  | Kenya | 105.83 | 75.36 | 92.16 | 8.77 | 0.10 | 0.70 | Hard Pegs |
|  | Tanzania | 2482.31 | 2173.71 | 2253.71 | 24.87 | 0.01 | 0.17 | Hard Pegs |
|  | Rwanda | 884.62 | 817.09 | 851.29 | 16.86 | 0.02 | 0.75 | Hard Pegs |
|  | Uganda | 3907.45 | 3556.70 | 3667.69 | 75.43 | 0.02 | 1.00 | Floating |
|  | Burundi | 1816.84 | 1511.71 | 1745.40 | 34.44 | 0.02 | 0.00 | Hard Pegs |
| **West Africa** | Guinea | 9505.94 | 8848.86 | 9083.53 | 133.50 | 0.01 | 0.00 | Other |
|  | Gambia | 49.72 | 38.61 | 46.73 | 1.48 | 0.03 | 1.00 | Other |
|  | Ghana | 4.84 | 2.72 | 4.03 | 0.52 | 0.13 | 0.00 | Floating |
|  | Togo | 628.59 | 523.76 | 568.34 | 27.12 | 0.05 | 0.17 | Hard Pegs |
|  | Nigeria | 324.50 | 147.10 | 200.96 | 64.88 | 0.32 | 0.30 | Hard Pegs |
|  | Senegal | 628.23 | 524.42 | 568.39 | 27.24 | 0.05 | 0.17 | Hard Pegs |
|  | Sierra Leone | 8420.37 | 5475.68 | 7529.03 | 516.00 | 0.07 | 0.17 | Other |
|  | Cote d'Ivoire | 628.23 | 524.42 | 568.39 | 27.24 | 0.05 | 0.17 | Hard Pegs |
|  | Rep. of Congo | 9860.09 | 5996.33 | 7912.89 | 906.63 | 0.11 | 0.17 | Hard Pegs |
|  | Gabon | 629.38 | 523.76 | 568.14 | 26.86 | 0.05 | 0.17 | Hard Pegs |
|  | Cameroon | 629.38 | 523.76 | 568.14 | 26.86 | 0.05 | 0.17 | Hard Pegs |
| **North Africa** | Algeria | 118.88 | 73.30 | 95.41 | 15.51 | 0.16 | 0.17 | Other |
|  | Egypt | 19.33 | 6.90 | 11.66 | 4.98 | 0.43 | 0.17 | Floating |
|  | Libya | 34.74 | 27.22 | 31.15 | 1.92 | 0.06 | 0.17 | Hard Pegs |
|  | Tunisia | 2.88 | 1.31 | 1.86 | 0.41 | 0.22 | 0.17 | Floating |
|  | Mauritania | 368.37 | 352.10 | 358.17 | 3.23 | 0.01 | 0.17 | Hard Pegs |
|  | Sudan | 47.62 | 2.37 | 8.41 | 6.42 | 0.76 | 0.30 | Hard Pegs |
| **Central and Eastern Europe** | Poland | 4.26 | 2.65 | 3.40 | 0.38 | 0.11 | 0.70 | Floating |
|  | Czech Rep. | 26.07 | 16.27 | 21.17 | 2.51 | 0.12 | 1.00 | Hard Pegs |
|  | Slovakia | 0.96 | 0.67 | 0.81 | 0.07 | 0.09 | 0.75 | Unkown |
|  | Hungary | 299.71 | 177.69 | 243.29 | 32.22 | 0.13 | 1.00 | Floating |
|  | Austria | 0.96 | 0.67 | 0.81 | 0.07 | 0.09 | 1.00 | Floating |
|  | Estonia | 15.03 | 10.72 | 12.76 | 1.15 | 0.09 | 1.00 | Floating |
|  | Latvia | 0.68 | 0.48 | 0.57 | 0.05 | 0.10 | 1.00 | Floating |
|  | Lithuania | 2.84 | 2.47 | 2.59 | 0.09 | 0.04 | 0.82 | Hard Pegs |
|  | Ukraine | 30.01 | 7.99 | 21.48 | 6.74 | 0.31 | 0.00 | Floating |
| **Southern Africa** | Zambia | 5385.22 | 5008.44 | 5245.07 | 59.87 | 0.01 | 1.00 | Floating |
|  | Zimbabwe | 16.88 | 6.57 | 10.74 | 2.74 | 0.25 | 0.42 | Other |
|  | Angola | 309.35 | 63.21 | 151.71 | 51.26 | 0.34 | 0.00 | Hard Pegs |
|  | South Africa | 16.88 | 6.57 | 10.74 | 2.74 | 0.25 | 0.17 | Floating |
|  | Namibia | 15.15 | 11.54 | 13.18 | 0.79 | 0.06 | 0.17 | Hard Pegs |
| **South Pacific** | New Zealand | 1.60 | 1.13 | 1.33 | 0.11 | 0.08 | 1.00 | Floating |
|  | Papua New Guinea | 3.37 | 1.97 | 2.78 | 0.43 | 0.16 | 0.47 | Hard Pegs |
|  | Samoa | 2.63 | 2.43 | 2.54 | 0.04 | 0.02 | 0.17 | Hard Pegs |
| **South Europe** | Serbia | 118.99 | 66.64 | 93.23 | 13.88 | 0.15 | NA | Hard Pegs |
|  | Croatia | 7.27 | 4.96 | 6.07 | 0.58 | 0.10 | 0.70 | Hard Pegs |
|  | Montenegro | 0.96 | 0.67 | 0.81 | 0.07 | 0.09 | NA | Soft Pegs |
|  | Slovenia | 0.96 | 0.67 | 0.81 | 0.07 | 0.09 | 0.70 | Floating |
|  | FYR Macedonia | 59.32 | 41.33 | 49.85 | 4.58 | 0.09 | 0.45 | Hard Pegs |
|  | Romania | 4.35 | 2.74 | 3.59 | 0.40 | 0.11 | 1.00 | Floating |
|  | Bulgaria | 1.89 | 1.31 | 1.58 | 0.15 | 0.09 | 1.00 | Soft Pegs |
|  | Albania | 133.35 | 95.21 | 111.24 | 9.47 | 0.09 | 0.42 | Floating |
|  | Greece | 0.96 | 0.67 | 0.81 | 0.07 | 0.09 | 0.75 | Floating |
|  | Malta | 0.96 | 0.67 | 0.81 | 0.07 | 0.09 | 1.00 | Unkown |
|  | Bosnia and Herzegovina | 1.89 | 1.40 | 1.66 | 0.13 | 0.08 | 0.17 | Soft Pegs |
| **Caribbean area** | Dominica | 2.77 | 2.44 | 2.70 | 0.02 | 0.01 | 0.42 | Soft Pegs |
|  | Dominican | 50.24 | 33.38 | 43.01 | 4.02 | 0.09 | 0.42 | Hard Pegs |
|  | Trinidad and Tobago | 6.79 | 6.31 | 6.56 | 0.18 | 0.03 | 1.00 | Hard Pegs |
|  | Antigua and Barbuda | 2.77 | 2.44 | 2.70 | 0.02 | 0.01 | 0.00 | Soft Pegs |
|  | Grenada | 2.77 | 2.44 | 2.70 | 0.02 | 0.01 | 0.17 | Soft Pegs |
| **Central America** | CostaRica | 616.89 | 498.75 | 547.41 | 20.88 | 0.04 | 1.00 | Hard Pegs |
|  | EISalvador | 8.87 | 8.62 | 8.75 | 0.02 | 0.00 | 0.70 | Soft Pegs |
|  | Panama | 1.02 | 0.71 | 1.00 | 0.01 | 0.01 | 1.00 | Soft Pegs |
| **South America** | Venezuela | 248209.92 | 2.14 | 5137.80 | 24159.72 | 4.70 | NA | Other |
|  | Suriname | 7.81 | 3.30 | 5.35 | 1.99 | 0.37 | 0.17 | Other |
|  | Uruguay | 33.21 | 20.95 | 27.44 | 3.24 | 0.12 | 1.00 | Floating |
|  | Guyana | 210.30 | 205.03 | 207.84 | 1.37 | 0.01 | 1.00 | Hard Pegs |
|  | Chile | 732.53 | 448.36 | 572.17 | 80.21 | 0.14 | 0.70 | Floating |
|  | Bolivia | 7.08 | 6.27 | 6.93 | 0.04 | 0.01 | 0.45 | Hard Pegs |
