# Supplementary material for: The dynamic evolution of the characteristics of exchange rate risks in countries along “The Belt and Road” based on network analysis
Source: PLoS One. 2019 Sep 6;14(9):e0221874. doi: 10.1371/journal.pone.0221874 (PMC6730902; doi:10.1371/journal.pone.0221874)
Supplement: S2 Table — (DOCX) [file pone.0221874.s002.docx]

S2 Table. The statistical description of exchange rate correlation network about “The B & R” participants before “The B & R” Initiative

| **Ranking** | **Region** | **Weighted Degree** | **Module** | **rate** |
| --- | --- | --- | --- | --- |
| 1 | CzechRep. | 34.8852678 | 1 | 100.00% |
| 2 | Hungary | 34.7251692 | 0 | 99.54% |
| 3 | Tunisia | 34.650877 | 0 | 99.33% |
| 4 | Poland | 34.2837543 | 0 | 98.28% |
| 5 | Albania | 33.5422295 | 1 | 96.15% |
| 6 | Russia | 33.4554174 | 0 | 95.90% |
| 7 | Romania | 33.0851495 | 0 | 94.84% |
| 8 | India | 32.4045429 | 0 | 92.89% |
| 9 | Indonesia | 31.9272922 | 0 | 91.52% |
| 10 | Serbia | 31.8193546 | 0 | 91.21% |
| **11** | **Euro** | **31.7860901** | **1** | **91.12%** |
| 12 | Zimbabwe | 30.6980871 | 0 | 88.00% |
| 13 | SouthAfrica | 30.6980871 | 0 | 88.00% |
| 14 | SriLanka | 28.7654138 | 0 | 82.46% |
| 15 | Armenia | 27.703104 | 0 | 79.41% |
| 16 | Syria | 26.9240192 | 0 | 77.18% |
| 17 | Turkey | 25.1489395 | 0 | 72.09% |
| 18 | Belarus | 24.7548346 | 0 | 70.96% |
| 19 | Uzbekistan | 23.8646231 | 0 | 68.41% |
| 20 | Israel | 23.7582896 | 1 | 68.10% |
| 21 | the UK | 22.5911105 | 1 | 64.76% |
| 22 | Nigeria | 19.8804087 | 0 | 56.99% |
| 23 | Bangladesh | 18.9809928 | 0 | 54.41% |
| 24 | Venezuela | 18.3119471 | 0 | 52.49% |
| 25 | Kuwait | 17.8840966 | 1 | 51.27% |
| 26 | South Korea | 15.6373038 | 1 | 44.82% |
| 27 | Chile | 15.6221858 | 1 | 44.78% |
| 28 | Japan | 13.9125991 | 1 | 39.88% |
| 29 | Malaysia | 12.7933066 | 1 | 36.67% |
| 30 | Ethiopia | 10.6774266 | 0 | 30.61% |
| 31 | AntiguaandBarbuda | 9.08247231 | 0 | 26.04% |
| 32 | Dominica | 9.08247231 | 0 | 26.04% |
| 33 | Grenada | 9.08247231 | 0 | 26.04% |
| 34 | Thailand | 8.88923286 | 1 | 25.48% |
| 35 | VietNam | 8.57922953 | 0 | 24.59% |
| 36 | SaudiArabia | 5.67852702 | 0 | 16.28% |
| 37 | Bahrain | 4.25523731 | 1 | 12.20% |
| 38 | UAE | 2.48086051 | 0 | 7.11% |
| 39 | Jordan | 0.41611508 | 0 | 1.19% |
| 40 | Singapore | 0.06711236 | 1 | 0.19% |
| 41 | Kenya | -0.8913064 | 0 | -2.55% |
| 42 | Panama | -1.1245573 | 1 | -3.22% |
| 43 | NewZealand | -2.6817111 | 1 | -7.69% |
| 44 | Philippines | -8.8172419 | 1 | -25.27% |
| 45 | Georgia | -9.730991 | 1 | -27.89% |
| 46 | Azerbaijan | -15.804859 | 1 | -45.31% |
| **47** | **China** | **-20.574499** | **1** | **-58.98%** |
| 48 | Qatar | -24.887524 | 1 | -71.34% |
| Average weighted degree | | 15.589 | | |
