# Supplementary material for: The dynamic evolution of the characteristics of exchange rate risks in countries along “The Belt and Road” based on network analysis
Source: PLoS One. 2019 Sep 6;14(9):e0221874. doi: 10.1371/journal.pone.0221874 (PMC6730902; doi:10.1371/journal.pone.0221874)
Supplement: S3 Table — (DOCX) [file pone.0221874.s003.docx]

S3 Table. The statistical description of exchange rate correlation network about “The B & R” participants after “The B & R” Initiative

| **Ranking** | **Region** | **Weighted Degree** | **Module** | **rate** |
| --- | --- | --- | --- | --- |
| 1 | Kuwait | 54.7011947 | 2 | 100.00% |
| 2 | Romania | 54.4961655 | 2 | 99.63% |
| 3 | Armenia | 53.9511205 | 2 | 98.63% |
| 4 | Malaysia | 53.7745725 | 2 | 98.31% |
| 5 | Georgia | 53.720715 | 0 | 98.21% |
| 6 | Kenya | 53.2052308 | 0 | 97.27% |
| 7 | Hungary | 53.1590087 | 2 | 97.18% |
| 8 | Russia | 52.8010158 | 2 | 96.53% |
| 9 | Singapore | 52.689026 | 2 | 96.32% |
| **10** | New Zealand | 52.0388466 | 2 | 95.13% |
| 11 | Indonesia | 51.7623728 | 0 | 94.63% |
| 12 | Poland | 51.5512439 | 2 | 94.24% |
| 13 | Chile | 51.333458 | 2 | 93.84% |
| **14** | **Euro** | **50.2721612** | **2** | **91.90%** |
| 15 | Serbia | 49.2805733 | 2 | 90.09% |
| 16 | VietNam | 49.1032283 | 0 | 89.77% |
| 17 | Azerbaijan | 47.985628 | 0 | 87.72% |
| 18 | Zimbabwe | 47.893627 | 2 | 87.55% |
| 19 | South Africa | 47.893627 | 2 | 87.55% |
| 20 | India | 47.7179762 | 0 | 87.23% |
| 21 | the UK | 47.1165139 | 0 | 86.13% |
| 22 | Tunisia | 46.9044157 | 0 | 85.75% |
| **23** | **China** | **44.8719209** | **0** | **82.03%** |
| 24 | SriLanka | 44.6109907 | 0 | 81.55% |
| 25 | South Korea | 44.0076183 | 2 | 80.45% |
| 26 | Nigeria | 42.7271044 | 0 | 78.11% |
| 27 | Philippines | 41.967873 | 0 | 76.72% |
| 28 | Turkey | 41.3217024 | 0 | 75.54% |
| 29 | Czech Rep. | 40.6069677 | 2 | 74.23% |
| 30 | Syria | 38.6673227 | 0 | 70.69% |
| 31 | Albania | 36.5271018 | 2 | 66.78% |
| 32 | Ethiopia | 35.5813916 | 0 | 65.05% |
| 33 | Thailand | 33.8351582 | 2 | 61.85% |
| 34 | Japan | 29.6436088 | 2 | 54.19% |
| 35 | Bangladesh | 29.618536 | 0 | 54.15% |
| 36 | Uzbekistan | 25.4883404 | 0 | 46.60% |
| 37 | Israel | 23.9335825 | 2 | 43.75% |
| 38 | Jordan | 22.660579 | 1 | 41.43% |
| 39 | Venezuela | 11.3685986 | 0 | 20.78% |
| 40 | Panama | 8.16399326 | 1 | 14.92% |
| 41 | Antigua and Barbuda | 6.08743265 | 1 | 11.13% |
| 42 | Dominica | 6.08743265 | 1 | 11.13% |
| 43 | Grenada | 6.08743265 | 1 | 11.13% |
| 44 | Bahrain | 2.22493762 | 2 | 4.07% |
| 45 | UAE | 1.18342399 | 0 | 2.16% |
| 46 | SaudiArabia | -3.2079787 | 2 | -5.86% |
| 47 | Belarus | -17.334239 | 2 | -31.69% |
| 48 | Qatar | -23.320683 | 2 | -42.63% |
| Average weighted degree | | 35.34920143 | | |

S1 Table 3. The statistical description of exchange rate correlation network about “The B & R” participants after “The B & R” Initiative
